# Results from the Canadian Nosocomial Infection Surveillance Program on Carbapenemase-Producing *Enterobacteriaceae*, 2010 to 2014

**DOI:** 10.1128/AAC.01359-16

**Published:** 2016-10-21

**Authors:** Laura F. Mataseje, Kahina Abdesselam, Julie Vachon, Robyn Mitchel, Elizabeth Bryce, Diane Roscoe, David A. Boyd, Joanne Embree, Kevin Katz, Pamela Kibsey, Andrew E. Simor, Geoffrey Taylor, Nathalie Turgeon, Joanne Langley, Denise Gravel, Kanchana Amaratunga, Michael R. Mulvey

**Affiliations:** a Antimicrobial Resistance and Nosocomial Infections, Public Health Agency of Canada, Winnipeg, MB, Canada; b Center for Communicable Diseases and Infection Control, Public Health Agency of Canada, Ottawa, ON, Canada; c Department of Pathology and Laboratory Medicine, Vancouver General Hospital, Vancouver, BC, Canada; d Department of Pediatrics and Child Health, University of Manitoba, Winnipeg, MB, Canada; e Department of Infection Prevention and Control, North York General Hospital, Toronto, ON, Canada; f Department of Laboratory Medicine, Victoria General Hospital, Victoria, BC, Canada; g Department of Infectious Diseases, Sunnybrook Health Sciences Centre, Toronto, ON, Canada; h Department of Infectious Diseases, University of Alberta Hospital, Edmonton, AB, Canada; i Department of Medical Microbiology, Hotel-Dieu de Quebec du CHUQ, QC, Canada; j Department of Pediatrics, IWK Health Centre, Halifax, NS, Canada

## Abstract

Carbapenemase-producing Enterobacteriaceae (CPE) are increasing globally; here we report on the investigation of CPE in Canada over a 5-year period. Participating acute care facilities across Canada submitted carbapenem-nonsusceptible Enterobacteriaceae from 1 January 2010 to 31 December 2014 to the National Microbiology Laboratory. All CPE were characterized by antimicrobial susceptibilities, pulsed-field gel electrophoresis, multilocus sequence typing, and plasmid restriction fragment length polymorphism analysis and had patient data collected using a standard questionnaire. The 5-year incidence rate of CPE was 0.09 per 10,000 patient days and 0.07 per 1,000 admissions. There were a total of 261 CPE isolated from 238 patients in 58 hospitals during the study period. *bla*_KPC-3_ (64.8%) and *bla*_NDM-1_ (17.6%) represented the highest proportion of carbapenemase genes detected in Canadian isolates. Patients who had a history of medical attention during international travel accounted for 21% of CPE cases. The hospital 30-day all-cause mortality rate for the 5-year surveillance period was 17.1 per 100 CPE cases. No significant increase in the occurrence of CPE was observed from 2010 to 2014. Nosocomial transmission of CPE, as well as international health care, is driving its persistence within Canada.

## INTRODUCTION

Gram-negative organisms account for 5 of the top 10 most common bacterial organisms isolated from patients in Canadian hospitals, with Enterobacteriaceae representing 33.8% of isolates (1). Enterobacteriaceae are implicated in both community- and hospital-acquired infections. Their plasticity and persistence can be attributed to the ease in which they are transferred (contaminated food, water, environmental surfaces, and hand carriage). The increase in carbapenem-resistant Enterobacteriaceae (CRE) is predominantly due to the acquisition of carbapenemase genes residing on mobile genetic elements (2).

Most carbapenemases hydrolyze penicillins, cephalosporins, and carbapenems. Additionally, carbapenemases are often associated with multidrug resistance, as they are commonly found on plasmids containing multiple determinants of resistance to other classes of antimicrobials, making treatment options limited and the use of antimicrobials such as tigecycline and colistin more common (3).

The occurrence of carbapenemase-producing Enterobacteriaceae (CPE) in Canada is rare. The first Canadian Nosocomial Infection Surveillance Program report on the occurrence of carbapenem-resistant Gram-negative bacilli from 2009 to 2010 indicated that only 0.1% of Enterobacteriaceae were resistant to carbapenems, and of those, 16.9% harbored a carbapenemase gene (4). In addition, in a national point prevalence survey, it was determined that CPE were identified in only 7% of Canadian hospitals in 2012 (5). Currently, Canadian reports of CPE from single hospital sites are becoming more common, including the emergence of *bla*_KPC-2/-3_ organisms among Klebsiella pneumoniae (6, 7) and more broadly among Enterobacteriaceae (8). Additionally, the emergence of *bla*_NDM-1_ (9, 10) and *bla*_OXA-48/-181_ (11, 12) organisms as well as outbreaks of *bla*_NDM-1_ (13) and *bla*_KPC-3_ (14) organisms have been described.

This report describes the incidence, risk factors for acquisition, epidemiology, and molecular mechanism of carbapenem-nonsusceptible Enterobacteriaceae over 5 years (2010 to 2014) from selected centers in a national hospital surveillance network.

## MATERIALS AND METHODS

### Surveillance period and surveillance population.

Surveillance was carried out between 1 January 2010 and 31 December 2014 and included in- and outpatients from Canadian acute care hospitals participating in the Canadian Nosocomial Infection Surveillance Program (CNISP). Participating hospitals increased from 33 in 2010 to 58 in 2014. The 58 CNISP hospitals were distributed among 10 Canadian provinces: 10 (17%) in the east (Prince Edward Island, New Brunswick, Nova Scotia, and Newfoundland and Labrador), 28 (48%) in central Canada (Quebec and Ontario), and 20 (35%) in the west (Manitoba, Saskatchewan, Alberta, and British Columbia).

### Study eligibility criteria.

Any Enterobacteriaceae collected from a patient (colonized or infected) admitted to a CNISP participating hospital, emergency department, or outpatient clinic that exhibited nonsusceptibility to imipenem, meropenem (≥2 mg/liter from 2010 to 2014), or ertapenem (≥0.5 mg/liter in 2010 to 2011 and ≥1 mg/liter in 2012 to 2014) in accordance with the Clinical and Laboratory Standards Institute (15) was considered eligible for inclusion. Primary laboratory detection and identification were conducted by either the hospital or the provincial laboratories using standard diagnostic laboratory procedures. All eligible isolates were sent to the National Microbiology Laboratory (NML; Winnipeg, Canada) for MIC confirmation using ertapenem, meropenem, and imipenem Etest strips (bioMérieux, St. Laurent, QC, Canada).

### Patient questionnaires.

The following were submitted with all isolates: age, sex, date of admission, date of positive culture, organism, ward, and anatomical site of isolation. From 2011 to 2014 a questionnaire was required for all patients with CPE which provided detailed patient history regarding travel, antimicrobial use, underlying medical conditions, and patient outcome. Patients were monitored for a maximum of 30 days after the date of the first positive CPE culture to determine all-cause deaths and deaths attributable to CPE infection (as judged by local case review).

### Molecular characterization of isolates.

Detection of the genes for the following carbapenemases by PCR was conducted as previously described (4): NDM, KPC, IMP, VIM, GES, and SME. Detection further included genes for OXA-48-type (16) and NMC/IMI-type (NMC-1 5′-TGGTGTCTACGCTTTAGAC-3′ NMC-2 5′-ACCATGTCTGATAGGTTTCC-3′) enzymes. PCR mapping of the Tn*4401* element was conducted using previously described primers (17). Multilocus sequence typing (MLST) (http://bigsdb.pasteur.fr/klebsiella/ and http://mlst.warwick.ac.uk/mlst/dbs/Ecoli) and macrorestriction analysis using pulsed-field gel electrophoresis (PFGE) as previously described (4) were conducted on all CPE. BioNumerics software (version 3.5; Applied Maths, Saint Lartens-Latem, Belgium) was used to analyze fingerprints. Plasmid restriction fragment length polymorphism (pRFLP) analysis was conducted as previously described using electroporation to transfer a carbapenemase gene-harboring plasmid to Escherichia coli DH10B (4) in which NDM-type plasmids were digested with BglII and all other carbapenemase gene-containing plasmids with EcoRI. Plasmid-based replicon typing (PBRT) was conducted as previously described (18, 19). Antimicrobial susceptibility testing was performed using Vitek2 (AST-GN25 or AST-N219; bioMérieux, St. Laurent, Canada) using 2014 CLSI breakpoints (20). Tigecycline breakpoints were based on FDA breakpoints for Enterobacteriaceae (susceptible [S], ≤2 mg/liter; intermediate, 4 mg/liter; resistant [R], ≥8 mg/liter). Etest (bioMérieux) was used to determine colistin MICs with EUCAST breakpoints for Enterobacteriaceae (S ≤ 2 mg/liter; R > 2 mg/liter). Antimicrobial testing on plasmids was done on representatives from large pRFLP clusters along with all plasmids with unique pRFLP profiles.

### Statistical analyses.

Annual incidence rates for CPE and CRE were calculated from the number of microorganisms tested divided by the number of patient days multiplied by 10,000 and number of microorganisms divided by patient admissions multiplied by 1,000. The numbers of microorganisms are provided by the submitting hospitals yearly. The numerators of these rates consists of the numbers of cases reported and therefore may include individuals more than once. Rates exclude cases identified in emergency departments and outpatient clinics.

Descriptive statistics and bivariate analysis were conducted on all molecular data. These data exceed the number of individual cases, as patients may have more than one microorganism identified. In addition, antimicrobial resistance rates were subjected to the Cochran-Armitage test in order to assess significance, at the 5% level, of year-related trends. Individual patient cases (inpatients, emergency patients, and outpatients) were used to derive patient demographics and risk factors (underlying condition, age, sex, type of carbapenemase, etc.). Comparisons were conducted using Fisher's exact two-sided test at the 5% significance level. Statistical analyses were performed using SAS EG version 5.1.

## RESULTS

### Patient demographics for CPE.

**FIG 1:**
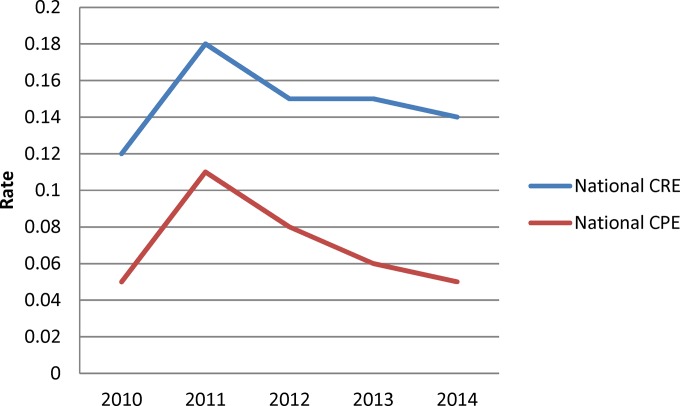
Overall national carbapenem-resistant Enterobacteriaceae (CRE) and carbapenemase-producing Enterobacteriaceae (CPE) rates per 1,000 patient admissions from 2010 to 2014.

A total of 823 carbapenem-intermediate or resistant Enterobacteriaceae were eligible for study inclusion. Of those, 613 (74.5%) were CRE and 261 (31.7%) were CPE as determined by PCR. Only 10 CPE (3.8%) were carbapenem intermediate by Etest, of which 7 produced KPC, 2 VIM, and 1 OXA-48. There were a total of 261 CPE isolated from 238 patients during the study period. From 2010 to 2014, the overall CNISP incidence rates of CRE and CPE per 10,000 patient days were 0.19 and 0.09 and per 1,000 admissions were 0.15 and 0.07, respectively (Fig. 1). Overall, the rates did not significantly increase over the 5 years. The increase in CRE and CPE in 2011 is most likely attributable to a KPC outbreak at a single hospital site.

Table 1 summarizes demographic data available for patients with CPEs. The majority of CPE cases (84%) were isolated from inpatients, while the remainder were identified from the outpatient setting (16%). Male patients contributed 61.9% of isolates. The most common sites of isolation were the rectum (48.7%), urine (26.1%), and respiratory tract (12.6%). Colonizations represented 59.4% of CPE cases. Although nosocomial transmission was observed in 55% of cases, approximately 80% of these cases were part of outbreaks at three hospital sites. Data on underlying medical conditions were available for 211 (88.7%) of the CPE cases, of which 89.1% (*n* = 188) included at least one underlying medical condition. The most commonly reported conditions were heart disease (36.7%), diabetes (29.8%), active cancer (25.5%), and lung disease (21.8%).

**TABLE 1:** Summary of available demographics from patients harboring carbapenemase-producing Enterobacteriaceae

Characteristic^*a*^	No. (%) of cases
Gender (*n* = 236)	
Male	146 (61.9)
Female	90 (38.1)
Age, yrs (*n* = 238)	
0–18	9 (3.8)
19–64	88 (37.0)
≥65	141 (59.2)
Hospital ward (*n* = 238)	
Medical	93 (39.1)
Emergency room	32 (13.4)
Intensive care unit	50 (21.0)
Surgical	34 (14.3)
Other	25 (10.5)
Isolate site (*n* = 238)	
Stool/rectal swab	116 (48.7)
Urine	62 (26.1)
Sputum	30 (12.6)
Blood	20 (8.4)
Skin/soft tissue	25 (10.5)
Surgical site	11 (4.6)
Other	9 (3.8)
Infections/colonizations (*n* = 212)	
Infection	86 (40.6)
Colonization	126 (59.4)
Nosocomial transmission (*n* = 164)	
Yes	91 (55.5)
No	73 (44.5)

a Demographic denominators vary depending on data available from hospital sites. Patients can report multiple hospital wards and isolation sites, which explains why the number of isolation site exceeds the number of patients. Patients can report organisms which can represent either infections or colonizations. However, cases that reported both a colonization and an infection would be classified only as infection, since infection is considered more important than colonization.

Data on international travel were collected from 201 (84.5%) patients with CPE cases. Table 2 describes the 49 cases (24.3%) involving persons who reported international travel within the 12 months prior to diagnosis; India was the most common travel destination, associated with 15 of the 49 cases (30.6%). Among these cases, 42 (85.7%) individuals reported that they had sought medical care while on international travel. Of the *bla*_NDM-type_-associated cases involving known travel, 87.5% were linked to patients who had sought medical attention within the Indian subcontinent. Similarity, 75% of *bla*_OXA-48-type_-associated cases involved patients who had sought medical attention in India.

**TABLE 2:** Distribution of CPE isolated from patients with international travel history within 12 months of date of positive culture

Country	Carbapenemase(s)	Organism(s)	No. of cases	Medical attention received^*a*^
Bahamas	KPC-2	K. pneumoniae	1	Y
Bangladesh	NDM-7	E. coli	1	Y
China, Shanghai	KPC-	K. pneumoniae	1	Y
Croatia	VIM-1	Enterobacter spp.	1	Y
United States	KPC-3	K. pneumoniae	1	Unknown
		Enterobacter spp.	2	Y
Ecuador	KPC-2	Enterobacter cloacae	1	Unknown
Egypt	OXA-48	E. coli	1	Y
Greece	KPC-2, VIM-1	K. pneumoniae	1	Y
	KPC-2	K. pneumoniae	1	Y
India	NDM-1	Providencia rettgeri	1	Y
		K. pneumoniae	4	Y (3), N (1)
		E. coli	1	Y
		Enterobacter spp.	1	Y
	NDM-5	E. coli	1	Y
	NDM-7	E. coli	2	Y
	OXA-181	K. pneumoniae	2	Y (1), N (1)
		E. coli	1	Y
	OXA-232	K. pneumoniae	1	Y
	NDM-1, OXA-232	K. pneumoniae	1	Y
Israel	KPC-3	K. pneumoniae	1	Y
	KPC-2	K. pneumoniae	1	Y
Italy	KPC-3	K. pneumoniae	1	Y
Oman	NDM-1	K. pneumoniae	1	Y
Pakistan	NDM-2	K. pneumoniae	1	Y
Puerto Rico	KPC-3	K. pneumoniae	1	Y
Saudi Arabia	OXA-48	K. pneumoniae	1	Y
		E. coli	1	Y
Serbia	NDM-1, OXA-48	K. pneumoniae	1	Y
Sri Lanka	NDM-1	K. pneumoniae	1	Y
Not specified	KPC-3	Enterobacter spp.	1	Unknown
		K. pneumoniae	2	Unknown (1), Y (1)
		Kluyvera spp.	1	Unknown
	NDM-type	K. pneumoniae	3	Y
		Enterobacter spp.	1	Y
		Providencia rettgeri	1	Y
		E. coli	1	Y
		S. marcesens	1	Unknown
	OXA-48	K. pneumoniae	1	Y
	OXA-181	K. pneumoniae	1	Unknown

a Y, yes; N, no.

Treatment data were available for 147 CPE cases (61.8%) and were collected only from 2010 to 2013. Antimicrobial treatment was administered to 73.5% (*n* = 108) of CPE cases within 2 weeks of their diagnosis. The most commonly prescribed antimicrobials were glycopeptides (29.5%), β-lactams (32%), fluoroquinolones (22.1%), and carbapenems (19.7%). Forty-nine percent of patients received >1 antimicrobial.

Thirty-day outcomes were available for 82 cases, with 14 (17.1%) deaths reported. Of total bloodstream infections (*n* = 19), nine (47.4%) deaths were reported. Death was reported as attributable to a CPE infection in four (28.6%) cases; three were bacteremic events (S. marcescens harboring *bla*_GES-5_ and K. pneumoniae and Enterobacter spp. harboring *bla*_KPC-3_) and one case involved a surgical site infection (K. pneumoniae harboring *bla*_OXA-232_).

### CPE.

**FIG 2:**
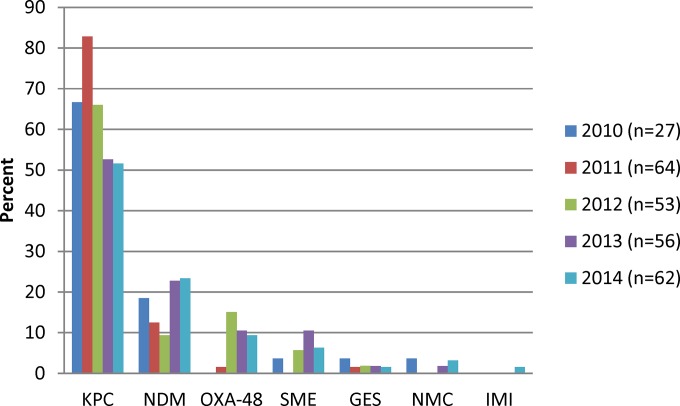
Proportion of carbapenemases by year (*n* = 265). Note that there were 261 CPE with 265 carbapenemases identified.

A total of 261 CPE were collected over the study period as follows: 2010, 26 (10.3%); 2011, 64 (24.5%); 2012, 53 (20.3%); 2013, 56 (21.5%); and 2014, 62 (23.8%). The proportion of carbapenemases observed per year is shown in Fig. 2. Over the 5 years of study, *bla*_KPC-type_ carbapenemases had the highest incidence, with an average of 66.9% of total CPE per year, followed by *bla*_NDM-1_ carbapenemases at 17.3% per year. Overall, there was a significant decrease in *bla*_KPC_ carbapenemases (*P* = 0.0458), whereas *bla*_SME_ (*P* = 0.0494) and *bla*_OXA-48-type_ (*P* < 0.0001) carbapenemases increased significantly.

All carbapenemases were sequenced to confirm variant type. There were 169 *bla*_KPC_ (156 *bla*_KPC-3_, 9 *bla*_KPC-2_, 2 *bla*_KPC-4_, and 2 unknown), 46 *bla*_NDM_ (36 *bla*_NDM-1_, 1 *bla*_NDM-3_, 2 *bla*_NDM-5_ 6 *bla*_NDM-7_, and 1 unknown), 21 *bla*_OXA-48-type_ (10 *bla*_OXA-48_, 9 *bla*_OXA-181_, and 2 *bla*_OXA-232_), 4 *bla*_VIM_ (3 *bla*_VIM-1_ and 1 *bla*_VIM-2_), 5 *bla*_GES-5_, 14 *bla*_SME_ (10 *bla*_SME-1_ and 4 *bla*_SME-2_), 1 *bla*_IMP-13_, and 4 *bla*_NMC/IMI_ (2 *bla*_NMC-A_, 1 *bla*_IMI-1_, and 1 *bla*_IMI-5_) carbapenemases. Four K. pneumoniae isolates contained multiple carbapenemases: 1 *bla*_KPC-2_ and *bla*_VIM-1_ carbapenemases, and 3 *bla*_NDM_ and *bla*_OXA-48-type_ carbapenemases. In addition, CPE coproduced the β-lactamases SHV (56.3%), TEM (49.0%), CTX-M (24.9%), OXA-1 (15.7%), and/or CMY-2 (8.0%). Table 3 describes the distribution of CPE among Enterobacteriaceae. K. pneumoniae, Enterobacter spp., and E. coli were the top three carbapenemase producers.

**TABLE 3:** Distribution of 261 CPE harboring 265 carbapenemases^*a*^

Pathogen	No. of isolates with indicated type of carbapenemase								Total
	NDM	KPC	OXA-48-type	VIM	GES	IMP	SME	NMC/IMI	
K. pneumoniae	28	88	15	1	0	0	NA	NA	132
E. coli	10	12	6	0	2	0	NA	NA	30
Enterobacter sp.	2	36	0	3	0	1	NA	4	46
Serratia sp.	1	14	0	0	3	0	14	NA	32
Citrobacter sp.	1	10	0	1	0	0	NA	NA	12
Klebsiella oxytoca	0	6	0	0	0	0	NA	NA	6
Morganella morganii	2	0	0	0	0	0	NA	NA	2
P. rettgeri	2	0	0	0	0	0	NA	NA	2
Pantoea sp.	0	1	0	0	0	0	NA	NA	1
Kluyvera sp.	0	2	0	0	0	0	NA	NA	2
Total	46	169	21	5	5	1	14	4	265

a Four isolates contained multiple carbapenemases; a K. pneumoniae isolate (bla_KPC-2_ and *bla*_VIM-1_) and three K. pneumoniae isolates (*bla*_NDM-1_ and *bla*_OXA-48_). NA, not applicable.

### Antimicrobial susceptibility testing of CPE.

Antimicrobial susceptibilities for all CPE over the 5 years of study can be found in Table S1 in the supplemental material. CPEs were commonly resistant to ciprofloxacin (59.8%), tobramycin (59.4%), and trimethoprim-sulfamethoxazole (70.1%). Resistance to tigecyline (18.4%) and colistin (5%) was observed. Over 5 years of study, CPEs demonstrated significantly increased resistance to piperacillin-tazobactam (*P* < 0.0001), cefazolin (*P* = 0.0094), meropenem (*P* = 0.0011), and gentamicin (*P* = 0.0336). There was a significant decrease in resistance to ciprofloxacin (*P* = 0.022), amikacin (*P* = 0.0031), and nitrofurantoin (*P* = 0.0029). These resistance patterns reflected the emergence of *bla*_OXA-48-type_ and *bla*_SME-type_ carbapenemase producers, which tended to have resistance to fewer non-β-lactam antimicrobials (Table 4).

**TABLE 4:** Antimicrobial resistance observed in CPE from 2010 to 2014

Antimicrobial	% of isolates producing indicated carbapenemase and resistant to antimicrobial							
	NDM (*n* = 43)	OXA-48 (*n* = 18)	KPC (*n* = 169)	GES (*n* = 5)	VIM (*n* = 4)	OXA-48 + NDM (*n* = 3)	SME (*n* = 14)	NMC (*n* = 4)
Ampicillin	100	100	100	100	100	100	100	100
Piperacillin-tazobactam	93	100	98.2	100	100	100	14.3	50
Cefazolin	100	100	100	100	100	100	100	75
Ceftriaxone	100	83.3	99.4	100	100	100	0	50
Ciprofloxacin	86	72.2	58.6	40	75	66.7	0	0
Ertapenem	100	100	96.4	100	75	100	100	100
Meropenem	90.7	61.1	88.8	100	75	100	92.9	75
Amikacin	60.5	11.1	29	20	25	66.7	0	0
Tobramycin	83.7	61.1	57.4	100	75	100	0	0
Gentamicin	76.7	72.2	33.1	80	50	100	0	0
Nitrofurantoin	79.1	72.2	55	80	50	66.7	100	0
Trimethoprim-sulfamethoxazole	76.7	77.8	72.8	80	100	100	0	0
Tigecylcine	18.6	22.2	20.1	0	0	66.7	0	0
Colistin	7	5.6	5.3	0	0	0	NA	0

Table 4 describes the antimicrobial resistance associated with each carbapenemase. Significantly higher rates of resistance were observed for ciprofloxacin (*P* < 0.001) and all aminoglycosides (*P* ≤ 0.001) in NDM producers than in KPC producers and for amikacin (*P* < 0.001) than in OXA-48-type producers. Nonsusceptibilities to tigecycline were observed in 47.3%, 43.5%, and 35.5% of *bla*_NDM_, *bla*_OXA-48-type_, and *bla*_KPC_ carbapenemase producers, respectively. Colistin resistance was observed in 7%, 5.6%, and 5.3% of *bla*_NDM_, *bla*_OXA-48-type_, and *bla*_KPC_ carbapenemase producers, respectively. One isolate, a K. pneumoniae ST512 *bla*_KPC_ carbapenemase producer, was resistant to all classes of antimicrobials tested, including tigecycline and colistin. A recent publication describes the interim recommendation of reporting extensively drug-resistant (XDRO) and pan-drug-resistant (PDRO) Gram-negatives (21). According to these recommendations, we found 122 (46.7%) and 53 (20.3%) of CPE to be XDRO and PDRO, respectively. The proportions of CPE with a PDRO phenotype were 41.0% of NDM, 16% of OXA-48-type, and 13% of KPC carbapenemase producers.

### DNA macrorestriction of CPE.

Pulsed-field gel electrophoresis (PFGE) was conducted on all carbapenemase-producing (CP) E. coli, Serratia marcescens, Enterobacter sp., and K. pneumoniae isolates.

Of the 46 CP Enterobacter spp., 36 (78.3%) produced *bla*_KPC-type_ enzymes. A single hospital site reported an ongoing outbreak that began in 2010 (14) and accounted for 4 KPC-3 clusters representing 41.3% of Enterobacter spp. (data not shown). The remaining Enterobacter spp. (15 producing *bla*_KPC-3_ carbapenemase, 1 *bla*_KPC-2_, 2 *bla*_KPC-4_, 2 *bla*_NDM-1_, 3 *bla*_VIM-1_, 1 *bla*_IMP-13_, and 3 *bla*_NMC/IMI_) all had unique pattern types and represent 11 different hospital sites.

K. pneumoniae isolates represented the majority of CPE (126/261 [48.3%]) and were shown to produce *bla*_KPC-type_ (*n* = 85), *bla*_NDM-1_ (*n* = 25), and *bla*_OXA-48-type_ (*n* = 12) carbapenemases as well as coproduce *bla*_KPC-2_ plus *bla*_VIM-1_ (*n* = 1) and *bla*_NDM-1_ plus *bla*_OXA-48-type_ (*n* = 3) carbapenemases (see Fig. S1 in the supplemental material). Of 126 CP K. pneumoniae isolates, 53.2% fall into 1/3 clonal clusters (I, *n* = 7; II, *n* = 48; and III, *n* = 12). Cluster I isolates are all sequence type 14 (ST14) isolates harboring *bla*_OXA-181_ (*n* = 5), *bla*_NDM-1_ (*n* = 1), or *bla*_NDM-1_ plus *bla*_OXA-252_ (*n* = 1) from either a western (*n* = 6) or central (*n* = 1) hospital site. Cluster II isolates are *bla*_KPC-3_ Tn*4401a*-like-carbapenemase-producing K. pneumoniae isolates mainly from a single hospital site in central Canada (38/48 [79.2%]). These isolates belonged to either ST258 or ST512 (a single base pair change in one allele from ST258). Cluster III isolates are *bla*_NDM-1_ carbapenemase-producing K. pneumoniae ST147 isolates from four sites in central Canada, one of which was associated with an outbreak due to nosocomial transmission in 2011 (13).

Among 30 CP E. coli (2 *bla*_GES-5_, 6 *bla*_OXA-48/-181_, 10 *bla*_NDM-1/-5/-7_, and 12 *bla*_KPC-3_) 28 unique PFGE fingerprints and 17 sequence types were observed (data not shown). The global endemic strain ST131 was observed in 5 *bla*_KPC-3_ carbapenemase-producing E. coli isolates.

Of 32 CP S. marcescens isolates identified, 12 (37.5%) clustered with >95% similarity (data not shown). These isolates were all *bla*_KPC-3_ carbapenemase producers that contained a Tn*4401b*-like element upstream of the *bla*_KPC-3_ gene. With one exception, all were from a single site in central Canada and were isolated in 2010 (*n* = 4), 2011 (*n* = 6), and 2012 (*n* = 2), suggesting a persistent *bla*_KPC-3_ carbapenemase-harboring clone at this site. A cluster of *bla*_SME-1_ carbapenemase producers (*n* = 5) were isolated from 3 hospital sites in western and central Canada between 2013 and 2014. This pulsotype has been previously observed across Canada (east, central, and west Canada), in 2010 to 2012 (22).

### Plasmid analysis.

Plasmids harboring carbapenemases were successfully isolated from 226 CP Enterobacteriaceae, and their pRFLP patterns were analyzed. Forty-one *bla*_NDM-1/3/5/7_-carrying plasmids were isolated (see Fig. S2 in the supplemental material). NDM plasmid patterns were mainly diverse, and only three small clusters were observed: a cluster with an unknown Inc group isolated from K. pneumoniae ST11, an IncR cluster from K. pneumoniae ST147 from an outbreak in 2011 (13), and a cluster with an unknown Inc group isolated from multiple species harboring *bla*_NDM-7_ observed in five different hospital sites across Canada. NDM plasmids also carried markers of resistance to aminoglycosides (82.5%) and trimethoprim-sulfamethoxazole (25%).

Of pRFLP patterns from 165 *bla*_KPC-type_, 4 *bla*_GES-5_, 3 *bla*_VIM-1_, and 11 *bla*_OXA-48-type_ carbapenemases, 101 (54.6%) fell into one of seven clusters (see Fig. S3 in the supplemental material). The largest cluster (II [*n* = 42]) belonged to IncFllA and consisted predominantly of K. pneumoniae (40/42 [95%]) *bla*_KPC-3_ producers. IncFllA plasmids contained additional markers of resistance to aminoglycosides (6.7%) and trimethoprim-sulfamethoxazole (20%). Though predominantly from a single hospital site (*n* = 32), these plasmids were also isolated from six other sites (*n* = 12). Conversely, the second largest cluster (IV [*n* = 24]) belonged to IncP and IncL/M, which all harbored *bla*_KPC-3_ and were isolated from 5 different bacterial species from a single hospital site. Plasmid type IncN (*n* = 50) was observed in several clusters (I, VI, and VII) as well as among numerous other pattern types. The IncN group has much more diverse pRFLP pattern types than the IncFllA or IncP/IncL/M groups. IncN plasmids were isolated from 6 different bacterial species, and all harbored *bla*_KPC-type_, with the exception of one *bla*_OXA-48_. IncN plasmids carried additional markers of resistance to the aminoglycosides (38.9%) and trimethoprim-sulfamethoxazole (86.1%).

Six of seven *bla*_OXA-48_ harboring plasmids clustered into an unknown incompatibility group (cluster V). These isolates came from 5 different hospital sites in 2012, 2013, and 2014, suggesting that a successful OXA-48 plasmid is circulating in Canada.

Isolation of IMI/NMC plasmids was attempted and not successful, suggesting chromosomal location of the genes.

## DISCUSSION

The global emergence of CPE is a growing concern. Although the first carbapenemase was described >20 years ago, extensive reporting of CPE has only occurred in the last 10 years (23). Reports of endemic KPC have been described from Latin America, the eastern United States, Europe, and Southeast Asia. In addition, the global dissemination of NDM since its discovery in 2009 (24) and the current emergence of OXA-48-type carbapenemase outside regions where it is endemic since its discovery in 2003 (16) have highlighted the global emergence and dissemination of CPE and the need for surveillance. In spite of increased global incidence of CRE, the current study from a national surveillance network in Canada reports a rate of 0.07 CPE/1,000 admissions or 0.09 CPE per 10,000 admissions, with no significant increase over the 5 years.

Limitations to the study include the lack of information regarding laboratory protocols for CPE screening and the criteria for screening patients for CPE. Some sites maybe underreporting CPE occurrence by identifying only clinical cases. A national survey on CPE laboratory practices and infection prevention surveillance is under way.

Common reservoirs have been described for specific carbapenemases: the United States, Israel, Greece, and Italy for *bla*_KPC_ carbapenemases; Turkey and North Africa for *bla*_OXA-48_ carbapenemases; and the Indian subcontinent is commonly associated with *bla*_NDM_, *bla*_KPC_, and *bla*_OXA-181_ carbapenemases (23). In the current study, we found that nearly a quarter (24.3%) of carbapenemases were associated with international travel, including health care. A large proportion of cases (48%) were first identified from rectal cultures performed for high-risk patient groups, reflecting the value of this type of screening in identifying CPE carriers. We found the *bla*_OXA-48_ and *bla*_NDM_ carbapenemase producers associated with patients that had traveled to India and sought medical attention, which is consistent with previous reports (25–27). Although *bla*_KPC_ carbapenemase producers were identified from a number of patients with history of travel to regions where *bla*_KPC_ carbapenemase producers are endemic (Greece, Italy, and the eastern United States), infection or colonization with *bla*_KPC_ carbapenemase producers was significantly less likely to be associated with patients who had traveled internationally than was infection or colonization with CPE producing either *bla*_NDM_ or *bla*_OXA-48-type_ carbapenemases (*P* < 0.0001). This is most likely attributed to a large outbreak of K. pneumoniae ST512 producing *bla*_KPC_ at a single hospital site over the course of this study (14).

Although *bla*_KPC_ represented the most common carbapenemase gene, there was a significant decrease in the number of *bla*_KPC_ carbapenemase producers over the study (*P* = 0.0458). This finding is most likely again due to the control of a large outbreak at a single site. Conversely, there was a significant increase in the number of cases involving *bla*_SME_ (*P* = 0.0494) and *bla*_OXA-48-like_ (*P* < 0.0001) carbapenemase producers. *bla*_SME_ was the first carbapenemase gene identified in a member of the Enterobacteriaceae originally isolated from a patient in London in 1982 (28). Current cases of organisms carrying *bla*_SME_ are only sporadically reported. The emergence of organisms carrying *bla*_SME_ in Canada is interesting, as there was no travel history associated with the cases and little similarity in macrorestriction patterns, making the potential reservoir for these cases unclear.

Higher mortality rates have been described for CPE infections, with inappropriate antimicrobial therapy suggested as a contributing factor (29). The current study revealed an all-cause mortality rate of 17.1/100 CPE infections, with 47.4% of bacteremias resulting in death. Most of the patients had multiple comorbidities, and attributable mortality could not be determined.

Molecular characterization of CPE revealed a great diversity in Canada not only in the types of carbapenemases but also in the species diversity and molecular subtypes. However, in some cases, large clonal clusters were observed, specifically in K. pneumoniae; indeed, over half of the K. pneumoniae isolates fell into one of three major clonal clusters: ST14, ST258/512, and ST147. Our *bla*_OXA-181_ findings with K. pneumoniae ST14 are similar to findings in a report from Finland (30); however, ST14 has been well described for other CPEs, like *bla*_NDM-1_ producers from India, Sweden, and the United Kingdom (27, 31). All patients in this cluster that had indicated recent international travel had sought medical attention within the Indian subcontinent, where *bla*_NDM-1_ and *bla*_OXA-181_ have been well documented (26). The predominance of the ST258/512 clone in Canada is not surprising, as the association of *bla*_KPC_ with K. pneumoniae ST258 has been well documented and this organism has even been suggested as one of the most highly successful multiresistant nosocomial pathogens known to date (32). Although ST147 has been previously reported for *bla*_NDM-type_ carbapenemase producers (31), which is what we report in this study, historically this sequence type was commonly reported as harboring *bla*_CTX-M-15_ and then later *bla*_KPC-type_ (33–35) and may represent the transfer of *bla*_NDM-types_ plasmids to a current successful clone, similar to the success of *bla*_KPC_ in K. pneumoniae ST258.

From the data we have presented in this report, the dissemination of *bla*_NDM_ in Canada is complex and may represent multiple independent introductions as opposed to a successful plasmid or clonal strain dissemination. Conversely, the association of IncFllA plasmids harboring *bla*_KPC_ with the successful K. pneumoniae ST258/512 has been well documented (4, 36–39) and represented ∼30% of KPC plasmids in this study. The more recently observed plasmids IncP/IncL/M and IncN have a much more diverse host range than the IncFllA plasmids and may contribute to the spread of the *bla*_KPC-3_ and *bla*_OXA-48-type_ genes between species and represent the current status of KPC and OXA-48 dissemination in Canada.

Apart from travel (with or without health care), the main force driving the spread of organisms producing carbapenemases in Canada has shifted from clonal outbreaks which were observed at two hospital sites (13, 14) to an increase in cases that are not linked by clonal lineage and include multiple Enterobacteriaceae species. These nonclonal cases in Canada represent the spread of many carbapenemase genes due to successful plasmids. Notably, many CPE in Canada are detected through surveillance cultures rather than clinically driven testing. It is thus critical for infection control that hospitals have programs for CPE surveillance to rapidly identify these organisms to minimize the risk of transmission. In addition, CPE surveillance programs encourage antibiotic stewardship by providing the data required to optimize appropriate therapy, improve patient outcome, ensure cost-effective therapy, and reduce adverse effects associated with inappropriate antimicrobial use.

## Supplementary Material

Supplemental material
