# Supplementary material for: Results from the Canadian Nosocomial Infection Surveillance Program on Carbapenemase-Producing Enterobacteriaceae, 2010 to 2014
Source: Antimicrob Agents Chemother. 2016 Oct 21;60(11):6787–94. doi: 10.1128/AAC.01359-16 (PMC5075087; doi:10.1128/AAC.01359-16)
Supplement: Supplemental material [file supp_60_11_6787__index.html]

Supplemental material 

# Results from the Canadian Nosocomial Infection Surveillance Program on Carbapenemase-Producing *Enterobacteriaceae*, 2010 to 2014

## Supplemental material

- Supplemental file 1 -

  Table S1, Fig. S1-S3

  PDF, 569K
