# Supplementary material for: Results from the Canadian Nosocomial Infection Surveillance Program on Carbapenemase-Producing Enterobacteriaceae, 2010 to 2014
Source: Antimicrob Agents Chemother. 2016 Oct 21;60(11):6787–94. doi: 10.1128/AAC.01359-16 (PMC5075087; doi:10.1128/AAC.01359-16)

Supplementary Table 1. Antimicrobial susceptibilities from 2010-2014 for all carbapenemase producers

	CLSI breakpoint criteria %(no.)																		
	2010 (n=26)			2011 (n=64)			2012 (n=53)			2013 (n=56)			2014 (n=62)			Overall (n=261)			
Antimicrobial Agent	%S	%I	%R	%S	%I	%R	%S	%I	%R	%S	%I	%R	%S	%I	%R	%S	%I	%R	MIC range (µg/mL)
Ampicillin	0	0	100	0	0	100	0	0	100	0	0	100	0	0	100	0	0	100	≥64
Piperacillin-Tazobactam	11.5	3.9	84.6	3.8	0	98.4	1.9	1.9	96.2	10.7	0	89.3	9.7	1.6	88.7	6.1	5.4	92.3	≤4-≥128
Cefazolin	3.9	0	96.2	0	0	100	0	0	100	0	0	100	0	0	100	0.4	0	99.6	≤1-≥64
Ceftriaxone	7.7	0	92.3	3.8	0	98.4	9.4	0	90.6	12.5	0	87.5	8.1	0	91.9	7.7	0	92.3	≤1-≥4
Ciprofloxacin	11.5	7.7	80.8	32.8	3.8	65.6	41.5	3.8	54.7	44.6	1.8	53.6	37.1	8.1	54.8	34.9	4.2	59.8	≤0.5-≥4
Ertapenem	0	0	100	0	3.8	98.4	3.8	0	96.2	3.6	0	96.4	1.6	1.6	96.8	1.9	0.8	97.3	≤0.5-≥8
Meropenem	7.7	11.5	80.8	14.1	9.4	76.6	9.4	1.9	88.7	3.6	3.6	92.9	3.2	1.6	95.2	7.7	5	87.4	≤1-≥4
Amikacin	42.3	0	15	64.1	3.1	32.8	71.7	1.9	26.4	67.9	1.8	30.4	74.2	3.2	22.3	66.7	2.3	31	≤16-≥64
Tobramycin	11.5	19.2	69.2	21.9	12.5	65.6	37.7	11.3	50.9	33.9	14.3	51.8	27.4	9.7	62.9	28	12.6	59.4	≤4-≥16
Gentamicin	61.5	11.5	26.9	46.9	14.1	39.1	50.9	5.7	43.4	51.8	3.6	44.6	41.9	6.5	51.6	49	8	42.9	≤4-≥16
Nitrofurantoin	3.9	7.7	88.5	15.6	18.8	65.6	24.5	17	58.5	25	12.5	62.5	27.4	21	51.6	21.1	16.5	62.5	≤32-≥128
Trimethoprim-Sulfamethoxazole	15.4	n/a	84.6	21.9	n/a	78.1	45.3	n/a	54.7	28.6	n/a	71.4	32.3	n/a	67.7	29.9	n/a	70.1	≤2/38-≥4/76
Tigecylcine	69.2	11.5	19.2	65.6	15.6	18.8	58.5	20.8	20.8	64.3	17.9	17.9	71	12.9	16.1	65.1	16.1	18.4	≤2-≥8
Colistin Etest[b]	69.2	n/a	7.7	79.7	n/a	6.3	92.5	n/a	1.9	76.8	n/a	5.4	87.1	n/a	4.8	83.1	n/a	5	0.047-≥256

Supplementary Figure 1. Pusled-field Gel Electrophoresis (PFGE) using *Xba*I of all Carbapenemase Producing *K. pneumoniae* from 2010-2014. Region-West (British Columbia, Alberta, Saskatchewan, Manitoba), Central (Ontario, Quebec) East (Nova Scotia, New Brunswick, Newfoundland, Prince Edward Island). ST-sequence type. Resistance Type: AMP-ampicillin, AMK-amikacin, AMC-amoxicillin/clavulanic acid, TZP-pipercillin/tazobactam, CFZ-cefazolin, FOX-cefoxitin, CPD-cefpodoxime ,CTX-cefotaxime, CAZ-ceftazidime, CRO-ceftriaxone, CIP-ciprofloxacin, ERT-ertapenem, MEM-meropenem, GEN-gentamicin, TOB-tobramycin, NIT-nitrofurantoin, TGC-tigecycline, SXT-trimethoprim/sulfamethoxazole, COL-colistin.

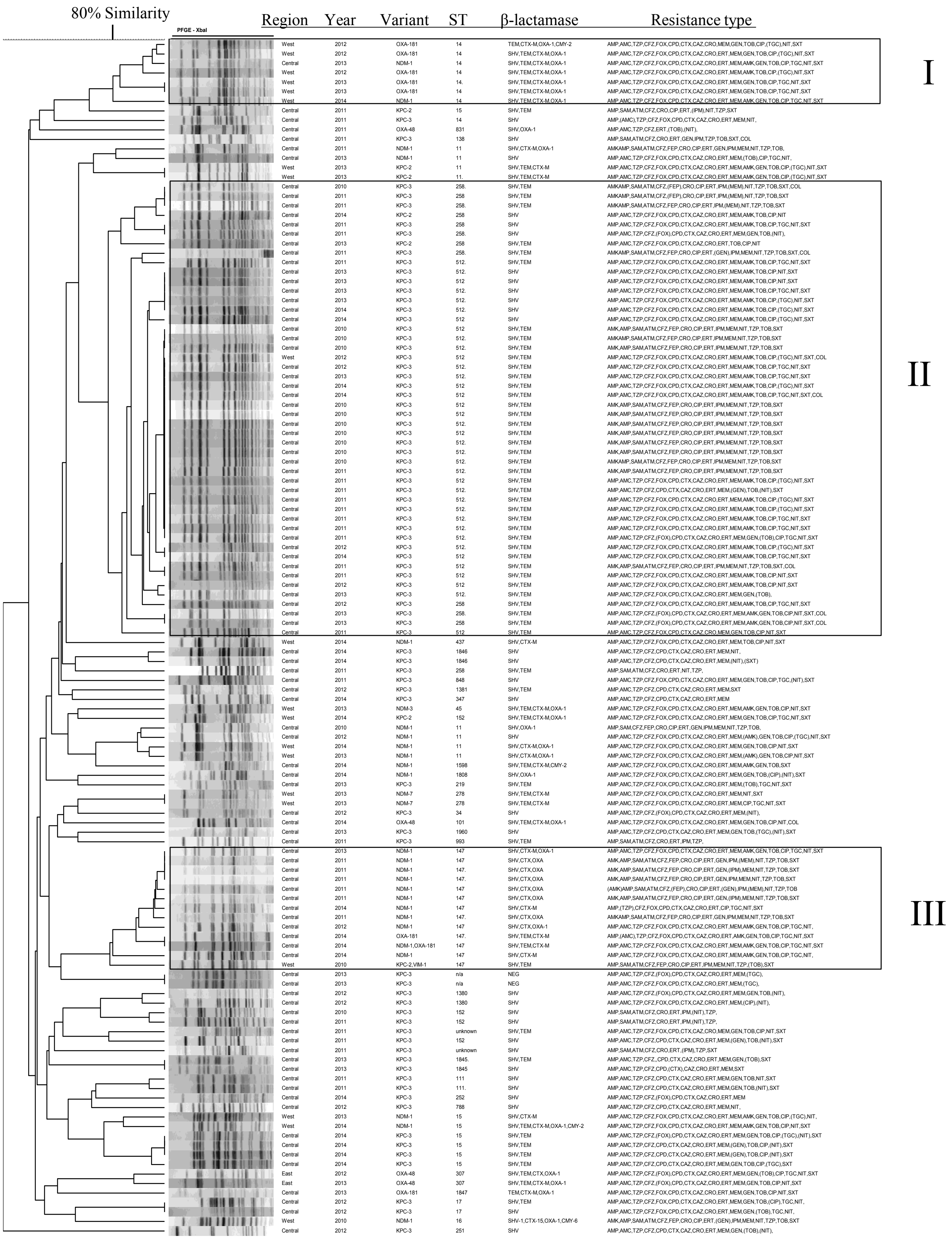

Supplementary Figure 2. Plasmid Restriction Fragment Length Polymorphisms (pRFLP) using BglII of 41 plasmids harbouring *bla*$_{\text{NDM-type}}$ from Enterobacteriaceae. Organism refers to the organism from which the plasmid was isolated. Region-West (British Columbia, Alberta, Saskatchewan, Manitoba), Central (Ontario, Quebec).RT-replicon type. Resistance type of plasmid: AMP-ampicillin, AMK-amikacin, AMC-amoxicillin/clavulanic acid, TZP-pipercillin/tazobactam, CFZ-cefazolin, FOX-cefoxitin, CPD-cefpodoxime ,CTX-cefotaxime, CAZ-ceftazidime, CRO-ceftriaxone, ERT-ertapenem, MEM-meropenem, GEN-gentamicin, TOB-tobramycin, SXT-trimethoprim/sulfamethoxazole.

80% Similarity

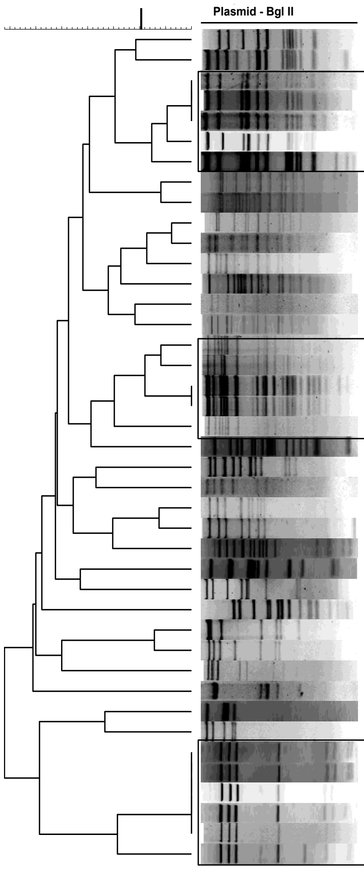

Organism	Year	Region	RT	Variant	Resistance type	
Klebsiella pneumoniae	2013	Central	R	NDM-1	AMP,AMC,TZP,CFZ,FOX,CPD,CTX,CAZ,CRO,ERT,MEM,AMK,GEN,TOB	
Klebsiella pneumoniae	2011	Central	R	NDM-1	AMK,AMP,SAM,CFZ,FEP,CRO,ERT,GEN,IPM,MEM,TZP,TOB,	
Klebsiella pneumoniae	2013	West	NEG	NDM-1	AMP,AMC,TZP,CFZ,FOX,CPD,CTX,CAZ,CRO,ERT,MEM,(AMK),GEN,TOB	
Klebsiella pneumoniae	2012	Central	NEG	NDM-1	AMP,AMC,TZP,CFZ,FOX,CPD,CTX,CAZ,CRO,ERT,MEM,AMK,GEN,TOB	
Klebsiella pneumoniae	2013	Central	NEG	NDM-1	AMP,AMC,TZP,CFZ,FOX,CPD,CTX,CAZ,CRO,ERT,MEM,AMK,GEN,TOB	I
Klebsiella pneumoniae	2014	West	NEG	NDM-1	AMP,AMC,TZP,CFZ,FOX,CPD,CTX,CAZ,CRO,ERT,MEM,AMK,GEN,TOB	
Klebsiella pneumoniae	2010	Central	NEG	NDM-1	AMK,AMP,SAM,CFZ,(FEP),CRO,ERT,GEN,IPM,MEM,TZP,TOB,	
Klebsiella pneumoniae	2010	West	A/C	NDM-1	AMK,AMP,AMC,CFZ,FEP,CRO,ERT,IMI,(MEM),TZP,GEN	
Escherichia coli	2010	West	A/C	NDM-1	AMK,AMP,AMC,CFZ,FEP,CRO,ERT,IMI,(MEM),TZP,GEN	
Escherichia coli	2012	Central	A/C	NDM-1	AMP,AMC,TZP,CFZ,FOX,CPD,CTX,CAZ,CRO,ERT,MEM,AMK,GEN,TOB,SXT	
Klebsiella pneumoniae	2014	Central	A/C	NDM-1	AMP,AMC,TZP,CFZ,FOX,CPD,CTX,CAZ,CRO,ERT,MEM,AMK,GEN,TOB	
Klebsiella pneumoniae	2014	West	A/C,R	NDM-1	AMP,AMC,TZP,CFZ,FOX,CPD,CTX,CAZ,CRO,ERT,MEM,AMK,GEN,TOB,SXT	
Enterobacter spp.	2012	Central	A/C	NDM-1	AMP,AMC,(TZP),CFZ,FOX,CPD,CTX,CAZ,CRO,ERT,(MEM),AMK,TOB,SXT	
Klebsiella pneumoniae	2014	Central	A/C	NDM-1	AMP,AMC,TZP,CFZ,FOX,CPD,CTX,CAZ,CRO,ERT,MEM,GEN,TOB,SXT	
Escherichia coli	2014	Central	A/C	NDM-1	AMP,AMC,TZP,CFZ,FOX,CPD,CTX,CAZ,CRO,ERT,MEM,GEN,TOB,SXT	
Klebsiella pneumoniae	2011	Central	R	NDM-1	AMK,AMP,SAM,CFZ,(FEP),CRO,ERT,GEN,IPM,MEM,TZP,TOB,	
Klebsiella pneumoniae	2011	Central	R	NDM-1	AMP,SAM,CFZ,(FEP),CRO,ERT,(GEN),IPM,MEM,(TZP),TOB,	
Klebsiella pneumoniae	2011	Central	R	NDM-1	AMK,AMP,SAM,CFZ,(FEP),CRO,ERT,GEN,IPM,MEM,TZP,TOB,	II
Klebsiella pneumoniae	2011	Central	R	NDM-1	AMK,AMP,SAM,CFZ,(FEP),CRO,ERT,GEN,IPM,MEM,TZP,TOB,	
Klebsiella pneumoniae	2012	Central	R	NDM-1	AMP,AMC,TZP,CFZ,FOX,CPD,CTX,CAZ,CRO,ERT,MEM,SXT	
Providencia rettgeri	2013	West	NEG	NDM-1	AMP,AMC,TZP,CFZ,FOX,CPD,CTX,CAZ,CRO,ERT,MEM,AMK,GEN,TOB,SXT	
Klebsiella pneumoniae	2013	Central	R	NDM-1	AMP,AMC,TZP,CFZ,FOX,CPD,CTX,CAZ,CRO,ERT,MEM,AMK,GEN,TOB,SXT	
Klebsiella pneumoniae	2014	Central	NEG	NDM-1	AMP,AMC,TZP,CFZ,FOX,CPD,CTX,CAZ,CRO,ERT,MEM,GEN,TOB	
Citrobacter spp.	2013	West	NEG	NDM-1	AMP,AMC,TZP,CFZ,FOX,CPD,CTX,CAZ,CRO,ERT,MEM,AMK,GEN,TOB	
Morganella morganii	2014	West	NEG	NDM-1	AMP,AMC,TZP,CFZ,FOX,CPD,CTX,CAZ,CRO,ERT,MEM	
Klebsiella pneumoniae	2013	West	NEG	NDM-3	AMP,AMC,TZP,CFZ,FOX,CPD,CTX,CAZ,CRO,ERT,MEM,AMK,GEN,TOB	
Providencia rettgeri	2010	Central	NEG	NDM-1	AMP,(AMC),TZP,CFZ,FOX,CPD,CTX,CAZ,CRO,ERT,MEM,AMK,GEN,TOB	
Klebsiella pneumoniae	2011	Central	NEG	NDM-1	AMP,SAM,CFZ,(FEP),CRO,ERT,IPM,MEM,TZP,	
Klebsiella pneumoniae	2011	Central	A/C	NDM-1	AMK,AMP,SAM,CFZ,(FEP),CRO,ERT,GEN,IPM,MEM,TZP,TOB,	
Escherichia coli	2014	West	L/M	NDM-1	AMP,AMC,TZP,CFZ,FOX,CPD,CTX,CAZ,CRO,ERT,MEM,GEN	
Enterobacter cloacae	2014	West	L/M	NDM-1	AMP,AMC,TZP,CFZ,FOX,CPD,CTX,CAZ,CRO,ERT,MEM,GEN	
Escherichia coli	2012	Central	FIA, FIB	NDM-1	AMP,AMC,CFZ,(FOX),CPD,CAZ,(CRO),(GEN),TOB	
Klebsiella pneumoniae	2014	West	NEG	NDM-1	AMP,AMC,TZP,CFZ,FOX,CPD,CTX,CAZ,CRO,ERT,MEM,(AMK),TOB,SXT	
Escherichia coli	2013	Central	FrepB	NDM-5	AMP,AMC,TZP,CFZ,FOX,CPD,CTX,CAZ,CRO,ERT,MEM,AMK,GEN,TOB,SXT	
Escherichia coli	2014	Central	FrepB	NDM-5	AMP,AMC,TZP,CFZ,FOX,CPD,CTX,CAZ,CRO,ERT,MEM,AMK,GEN,TOB,SXT	
Klebsiella pneumoniae	2013	West	NEG	NDM-7	AMP,AMC,TZP,CFZ,FOX,CPD,CTX,CAZ,CRO,ERT,MEM	
Klebsiella pneumoniae	2013	West	NEG	NDM-7	AMP,AMC,TZP,CFZ,FOX,CPD,CTX,CAZ,CRO,ERT,MEM	
Escherichia coli	2013	West	NEG	NDM-7	AMP,AMC,TZP,CFZ,FOX,CPD,CTX,CAZ,CRO,ERT,MEM	
Escherichia coli	2014	Central	NEG	NDM-7	AMP,AMC,TZP,CFZ,FOX,CPD,CTX,CAZ,CRO,ERT,MEM	III
Escherichia coli	2011	West	NEG	NDM-7	AMP,AMC,TZP,CFZ,FOX,CPD,CTX,CAZ,CRO,ERT,MEM	
Serratia marcescens	2013	West	NEG	NDM-7	AMP,AMC,TZP,CFZ,FOX,CPD,CTX,CAZ,CRO,ERT,MEM	

Supplementary Figure 3. Plasmid Restriction Fragment Length Polymorphisms (pRFLP) using EcoR1 of 111 plasmids harbouring carbapenemases from Enterobacteriaceae. Region-West (British Columbia, Alberta, Saskatchewan, Manitoba), Central (Ontario, Quebec). Organism refers to organism from which the plasmid was isolated. Resistant type of plasmid: AMP-ampicillin, AMK-amikacin, AMC-amoxicillin/clavulanic acid, TZP-pipercillin/tazobactam, CFZ-cefazolin, FOX-cefoxitin, CPD-cefpodoxime ,CTX-cefotaxime, CAZ-ceftazidime, CRO-ceftriaxone, ERT-ertapenem, MEM-meropenem, GEN-gentamicin, TOB-tobramycin, SXT-trimethoprim/sulfamethoxazole. Un-unique refers to a plasmid pattern indistinguishable from another, resistance type was not determined for those plasmids.

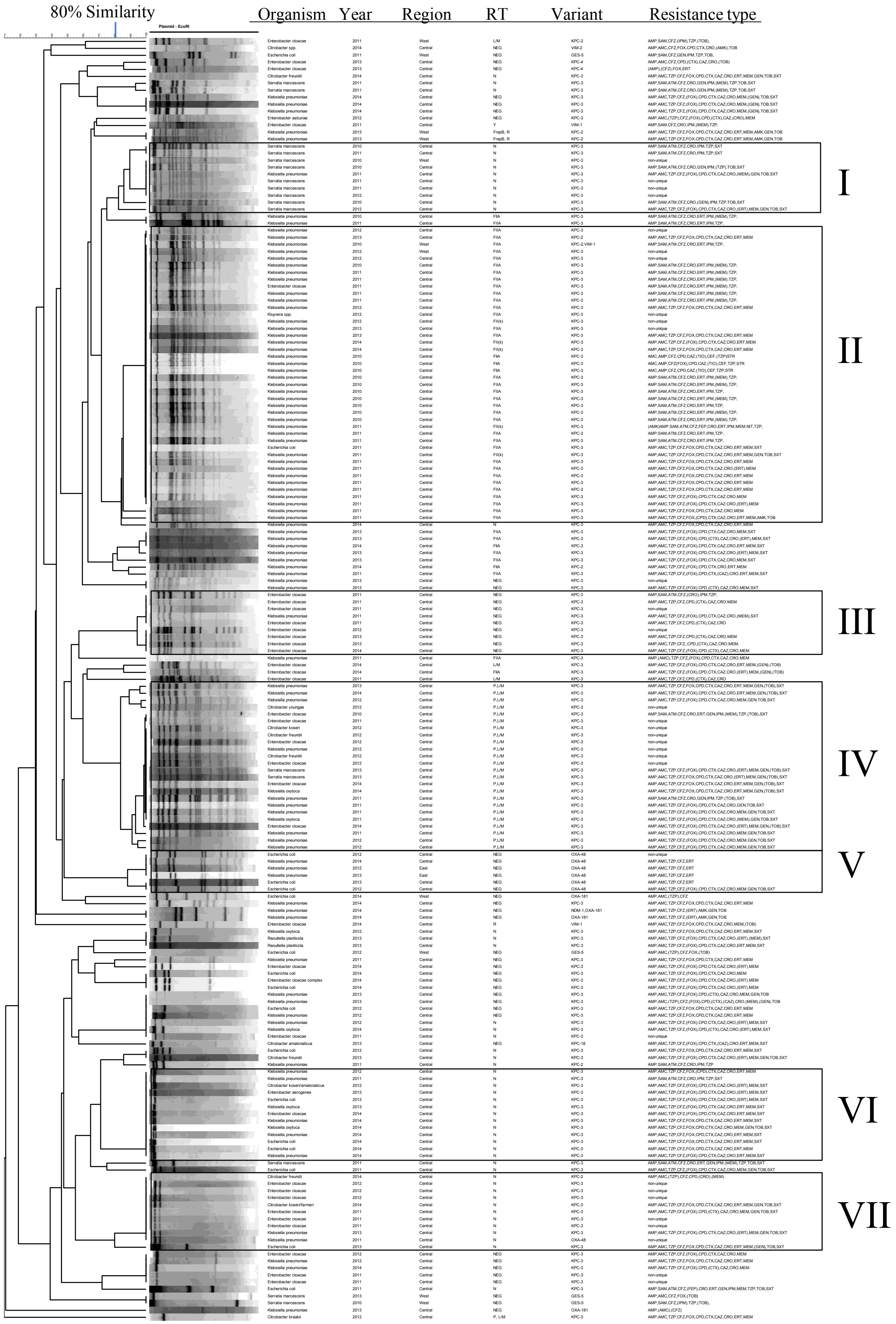

Supplement: Supplemental material [file AAC.01359-16_zac011165703so1.pdf]
